# Advanced tools and methods for single-cell surgery

**DOI:** 10.1038/s41378-022-00376-0

**Published:** 2022-04-29

**Authors:** Adnan Shakoor, Wendi Gao, Libo Zhao, Zhuangde Jiang, Dong Sun

**Affiliations:** 1 Department of Biomedical Engineering, City University of Hong Kong, Hong Kong, China; 2 State Key Laboratory for Manufacturing Systems Engineering, International Joint Laboratory for Micro/Nano Manufacturing and Measurement Technologies, The School of Mechanical Engineering, Xi’an Jiaotong University, Xi’an, China

**Keywords:** Engineering, Nanobiotechnology

## Abstract

Highly precise micromanipulation tools that can manipulate and interrogate cell organelles and components must be developed to support the rapid development of new cell-based medical therapies, thereby facilitating in-depth understanding of cell dynamics, cell component functions, and disease mechanisms. This paper presents a literature review on micro/nanomanipulation tools and their control methods for single-cell surgery. Micromanipulation methods specifically based on laser, microneedle, and untethered micro/nanotools are presented in detail. The limitations of these techniques are also discussed. The biological significance and clinical applications of single-cell surgery are also addressed in this paper.

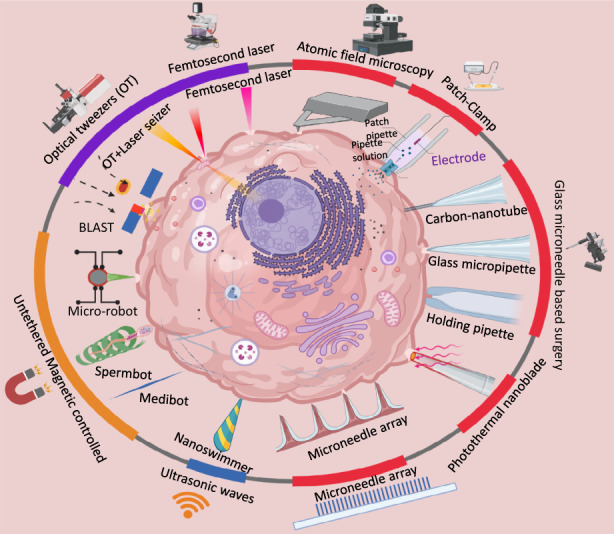

## Introduction

The molecular biology of individual cells must be explored to understand the normal physiology and functional mechanism of cells in response to disease or injury. Cellular functions, such as metabolism, cell motility, and gene expression, are greatly affected by the properties of intracellular structures and organelles. Technologies that can explore the genetic factors and molecules that contribute to disease evolution at a single-cell level have become increasingly important to elucidate the molecular basis underlying the proliferation, transformation and metastasis of cancer cells^1–4^. Research on whole tissues delivers only a statistical average of numerous activities occurring in different cells. There has been a paradigm shift in modern surgery and medicine, with technologies becoming smaller and more efficient than their larger predecessors, increasing their effectiveness^5^. This has led to major improvements in areas such as minimally invasive surgery, drug delivery, diagnostics, and cell manipulation^6^. Studies of single-cell manipulation and surgery may suggest that genetic changes activated by tumorigenesis-related signaling pathways can cause healthy cells to mutate, becoming cancer cells^7^. Therefore, micro/nanomanipulation methods and tools that can perform single-cell manipulation and surgery have attracted worldwide attention in recent years, as they provide information about individual cells and their organelles^8–13^.

There are numerous key applications of single-cell surgery, such as cloning^12^, preimplantation and diagnosis^14^, gene editing^15^, cellular therapy^16^, and understanding the functions and activities of subcellular organelles and components^7^. Several micro/nanomanipulation tools and systems for single-cell manipulation and surgery, such as glass microneedles^17–19^, optical tweezers (OTs)^20^, microfluidics^21^, atomic force microscopy (AFM)^22^, dielectrophoresis^23^, and magnetic tweezers^24^, have been used for manipulating and interrogating single cells. Glass microneedles can be fabricated by pulling glass capillaries on heating filaments. The resultant capillaries feature a conical shape with one end from a few micrometers to a few nanometers in diameter. Several programmable micropipette pulling instruments can create micropipette tips with different sizes and shapes depending on their application, such as force probes for investigating muscle physiology. Kishino and Yanagida^25^ fabricated a micropipette 0.3 μm in diameter at the tip and 70–100 μm in length from a 1 mm glass capillary by using an electrode micropipette puller. Optical tweezers are strongly focused laser beams that can be used as end-effector tools to trap and manipulate dielectric particles with sizes ranging between a few nanometers and a few micrometers^26^. Given their noninvasiveness, flexibility, and precise manipulation of objects, OTs have been applied to numerous biological tasks, such as the rotation, transportation, stretching, and assembly of cells. Microfluidics is an interdisciplinary methodology at the intersection of many fields, such as nanotechnology, micromechanics, analytical chemistry, bioengineering, and microelectronics. Microfluidics was invented in the early 1990s by Manz et al.^27^. The goal of microfluidic technologies is to create a platform with a size of a few square centimeters or less to simplify operations involved in biology and chemistry, such as sample separation, preparation, detection, sorting reaction, and lysis. Improvements in on-chip integration and soft lithography have enabled us to create a network of microchannels that can be used to understand cell biology at a single-cell level by controlling fluid at the microliter and picoliter scales.

The above techniques and tools can be integrated to achieve precise, versatile, and advanced cellular and subcellular manipulations. Compared to traditional chemical and biological methods for cell manipulation, these methodologies offer numerous advantages, such as control of single or multiple cells, accurate simultaneous manipulation, the ability to approach subcellular structures and organelles, and flexible and repeatable cell manipulation. Cell micromanipulation methods also play important roles in precise cell surgery. In general, micro/nanomanipulation tools for single-cell surgery can be divided into three types: laser-based, microneedle-based, and untethered-micro/nanotool-based methods. The following sections describe the details of tools and methods developed for single-cell surgery using lasers, microneedles, and untethered micro/nanorobots.

## Micro/nanomanipulation methods for single-cell surgery

**Fig. 1:**
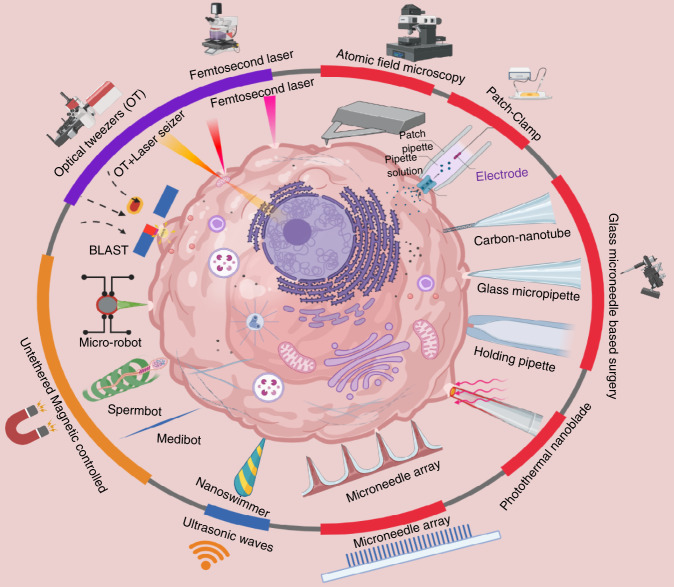
Single-cell surgery tools and methods. Examples of microneedles, lasers, and untethered microrobots based surgery tools are shown under red, purple, and orange patches, respectively.

In recent decades, several micro/nanomanipulation tools have been developed for micromanipulation tasks (Fig. 1)^28–38^. Specific mRNAs were extracted from live cells with AFM tips^39^, and the cells were punctured by nanorobots^39^. Carbon nanotubes attached at one end of a glass micropipette were used to transfer fluids in cells, interrogate cells, and perform electrochemical and optical diagnostics^40^. A number of laser systems have been used to perform surgery on cell membranes, including continuous wave and pulsed picosecond and nanosecond lasers^41^. Many other approaches use microscale devices, such as sampling probes^42,43^, semiclosed microchips^44^ and computer-assisted patch clamping^45^, to analyze cellular contents. The following subsections describe the details of the tools and methods developed for single-cell surgery using lasers, microneedles, and untethered microdevices.

### Laser-based single-cell surgery methods

**Fig. 2:**
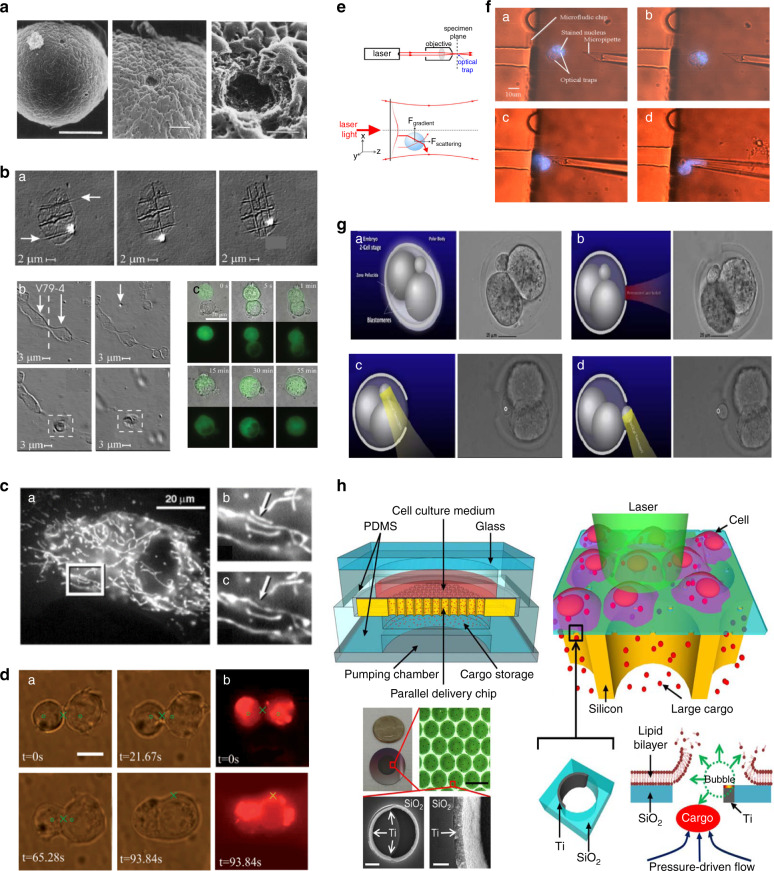
Laser-based single-cell surgery tools and methods. **a** SEM images after exposure to an argon fluoride excimer laser to drill holes in the zona pellucida of mouse oocytes. Adapted with permission^183^. Scale bars are 20, 5, and 2 µm (left to right). **b** (a) Membrane surgery on a live MDCK cell. Adapted with permission^50^. (b) Isolation of two live fibroblast cells. (c) Fusion of two hESCs in suspension^52^. **c** Depletion of single mitochondrion by a femtosecond laser in a live cell. Adapted with permission^51^. (b) Before laser irradiation. (c) After exposure to a femtosecond laser. **d** Fusion of cells by solid-state laser^185^. (a) Fusion of two Jurkat cells at various times. (b) Fluorescence pictures of the relative positions of the two nuclei before and after fusion. **e** Working phenomena of optical tweezers. A 3D light gradient is created when light arrives at the objective lens of a microscope, and microparticles can be held in the center of the trap. The gradient and scattering components of optical forces on microscale particles are controlled by a Gaussian laser beam^26^. **f** OT-assisted single-cell biopsy^21^. (a–c) Cell orientation control. (d) Biopsy with the help of a microneedle. **g** Snapshots of polar body biopsy in mouse embryos using a combined laser scalpel and OT system. Adapted with permission^57^. (a) A snapshot before surgery. (b) A snapshot after membrane ablation. (c) OT trapping. (d) Organelle extraction. **H** Schematic and image of a laser-based high-throughput cell injection system (BLAST). Copyright 2015, Springer Nature^67^.

Optical transfection, which uses light in the form of a focused laser to deliver drugs into cells, has also garnered scientific interest. Optics-based technology is also useful for manipulating biological materials at the micron and submicron scales^46^. Various laser types may generate small temporary holes that facilitate the transport of plasmid DNA and other macromolecules. Laser-based single-cell surgery methods provide a noncontact, rapid, and sterile way of introducing membrane-impermeable compounds into cells. High-speed laser pulses for single-cell surgery have been widely studied in the past few years^47,48^. A noninvasive argon fluoride excimer laser with a wavelength of 193 nm was used to drill a hole in the outer membrane (zona pellucida) of mouse oocytes, as shown in Fig. 2a^49^. Figure 2b shows the application of high-intensity femtosecond (fs) laser pulses to live mammalian cells to perform nanosurgical cell isolation and membrane surgery^50^. As a high-resolution ablation tool, fs lasers were also used to disrupt single organelles of cells, such as single mitochondria (Fig. 2c)^51^.

Chen et al. developed a method of cell fusion by cutting the cell membrane^52^, as shown in Fig. 2d. In their method, two cells were placed near each other, and the membranes of both cells at the common junction were carefully cut by a solid-state laser, which resulted in cell fusion. However, the efficiency of cell fusion by this laser ablation was very low.

The use of an ultrafast pulsed laser for optical transfection allows precise targeting and excellent viability^53,54^. Even though different optical technologies are continuously being developed, several issues remain with the intracellular delivery of genes/transcripts, such as low efficiency and repeatability. Combining optical transfection with microfluidic technology has widely been utilized to improve transfection efficiency and throughput. To accomplish optical reprogramming of large cell populations, Uchugonova et al. developed an ultrashort fs laser-microfluidic cell transfection device^55^. Ultrashort laser pulses cause temporary membrane permeabilization in a microfluidic tube containing several genes, allowing the generation of contamination-free induced pluripotent stem cells^55^.

Schomaker et al. presented an optical transfection technique based on photosensitive materials^56^. They cultured cells with gold nanoparticles (AuNPs) and subjected them to fs laser pulses. Laser stimulation can activate photosensitive molecules localized to the membranes of endocytic vesicles, resulting in localized membrane permeabilization of the cell^57^. Cell processing for gene and cell treatments typically employs numerous distinct methods for gene transfer and cell separation or removal. Lukianova-Hleb et al. pioneered the use of plasmonic nanobubbles (PNBs) to simultaneously transfect target cells while eliminating undesired subsets of other cells^58^. After brief laser irradiation, transient PNBs were produced around AuNPs, resulting in a nanoscale explosion that enabled molecular cargo transmembrane injection.

OTs have been effectively used to investigate numerous biological applications in recent years^21,59,60^. As demonstrated in Fig. 2e, OTs utilize a Gaussian laser beam, which can create a 3D light gradient when focused by an objective lens, to manipulate microscopic particles that face two types of forces: the gradient force introduced by a scattering force and the gradient of field intensity produced by the photons hitting the particle along their transmission direction^26^. This scattering force must be overcome by the gradient force along the optical axis to form a stable trap. Trapping of micrometer-sized transparent particles was introduced by Arthur Ashkin in 1970^61^ and then used to manipulate and trap bacteria and viruses^62^. OTs have also been exploited to perform numerous biological tasks, such as cell stretching and transportation^63,64^, mechanical property calibration^65^, and cell assembly^57^, because they can operate microparticles noninvasively, flexibly, and precisely. As the power of the OTs is in the range of a few hundred milliwatts in the sample plane, it is not sufficient to counteract the internal pressure of the cell or break the cell membrane to force external particles or internal organelles to move in or out of the cell, respectively. Therefore, OT has been used in conjunction with other micromanipulation tools to perform cell surgery. Two OTs were pointed on a single cell, and their position was controlled to alter the 3D position of the cell, which eventually made biopsies easier by bringing the nucleus opposite to the micropipette, as shown in Fig. 2f. Inna V. Il’ina et al. established a laser-based polar body biopsy method for preimplantation genetic diagnosis^57^. They used a fs laser to cut a hole in the membrane of an embryo and then utilized OTs to extract the polar body from the embryo, as shown in Fig. 2g. The removal of cell organelles from a yeast cell by OTs was presented in^66^. First, a fs laser was used to disrupt the cell membrane, and OTs were used to extract subcellular parts by changing the functioning mode of the laser. Similarly, we utilized OT to control the 3D orientation of a single cell prior to cell surgery, which greatly improved the efficiency of the surgical process^21^.

A laser-induced cavitation bubbled methodology (BLAST) was developed to increase the throughput of delivery of the particles in mammalian cells^67^. The abrupt generation of bubbles induces high stress on the cell membrane, which eventually creates pores in the membrane for intracellular delivery, as shown in Fig. 2h. Under rapid laser scanning, BLAST methodology can inject micron-sized particles into 100,000 cells in 1 min. The existing laser scissors methods, such as the fs laser methods described above, enable precise surgery in cells at the organelle level, but they are limited by throughput. High-throughput surgery methods such as BLAST can rapidly perform surgery on thousands of cells in parallel, but they fail to achieve organelle-level precision simultaneously. Achieving high precision and throughput of cell surgery at the same time is challenging but needed. Robotic control of laser scissors may enhance the speed of processed cells while maintaining precision levels.

The possible biological applications of laser-based cell surgery devices are broad. High-throughput laser-based surgical methods, such as BLAST, are now able to deliver ultralarge cargoes into relatively cells in a minimally invasive manner that was not previously possible with other methods^41,67^. For example, the delivery of mitochondria for the study of diseases caused by mutated mitochondrial DNA, the delivery of whole chromosomes for cell engineering, and the delivery of intracellular pathogens for the study of pathogenesis all become possible. Due to the massively parallel and near-simultaneous nature of delivery achieved by methods such as BLAST^67^, a single chip can be used to conduct experiments and generate enough data for statistical analysis. Researchers will be able to observe large numbers of infected cells over time to examine phenomena such as bacterial localization and intracellular proliferation due to the ability to transfer bacteria into 100,000 host cells at a time. Such studies are practically difficult to perform using standard pipette-based delivery systems, as they do not provide the throughput required for accurate statistical analysis, and rapid events do not benefit from synchronization due to coinfection.

### Micro/nanoneedle-based single-cell surgery methods

**Fig. 3:**
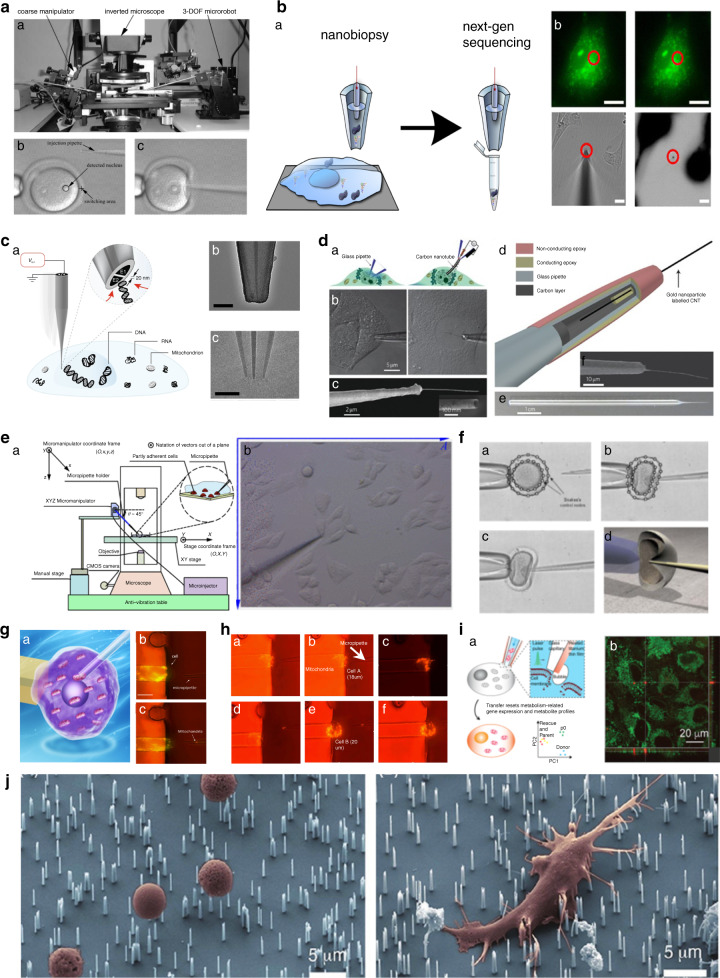
Micro/nanoneedle-based single-cell surgery tools and methods. **a** (a) Autonomous embryo injection system. Adapted with permission^75^. (b) An embryo is held by a holding micropipette, and DNA injection is performed with a microneedle in its pronuclei. Adapted with permission^75^. **b** Scanning ion conductance microscopy for nano-biopsy. Adapted with permission^78^. (a) Schematic of the nanobiopsy procedure. (b) Fluorescence images of biopsy (upper) and release of mitochondria (lower). **c** (a) Schematic of DEP nanotweezers. Copyright 2018, Springer Nature^79^. b) Transmission electron microscopy micrographs of DEP nanotweezers before (left) and after (right) carbon deposition. Scale bars are 100 nm. **d** Nanotube-based cellular endoscope. Copyright 2010, Springer Nature^40^. (a) Comparison between cellular endoscopes and glass pipettes. (b) A HeLa cell is injected with a commercial glass pipette (left), and the rat hepatocyte nucleus is examined with a 100 nm nanotube endoscope (right). (c) SEM image of an assembled endoscope with a carbon nanotube tip of 100 nm. (d) Schematics of the nanotube endoscope. (e) Optical image of a carbon nanotube-tipped glass pipette. (f) SEM images of the assembled endoscope with carbon nanotube tips of 50 nm. **e** Single-cell injection by robotically controlled microneedle^18^. (a) Schematics of the system. (b) Experimental photograph of single-cell injection by microneedle. **f** Biological cell injection with a glass microneedle controlled by an augmented human–machine interface system. Copyright 2015, IEEE^186^. **g** Automatic mitochondrial biopsy system^19^. (a) Schematic of the mitochondrial biopsy system. (b) Before the biopsy of mitochondria from a single cell with a microneedle and (c) after mitochondrial biopsy. **h** Automatic mitochondrial transfer from a single cell to another single cell^80^. (a–c) Steps of mitochondrial extraction. (d–f) Steps of mitochondrial transfer. **i** Photothermal nanoblade transfer of isolated mitochondria. Adapted with permission^85^. (a) Schematic of photothermal nanoblade mitochondrial transfer. (b) Confocal microscopy image of mitochondrial transfer with photothermal nanoblades. **j** SEM images of B cells (left) and dendritic cells (right) on top of NWs. Adapted with permission^184^.

Intracellular surgery using microneedles dates back to the work of Barber et al., who injected living cells with substances such as bacteria by using a very tiny glass needle loaded with injection solutions^68^. Glass microneedles, which can be fabricated by pulling glass capillaries on heating filaments, have been used for single-cell manipulation and surgery in recent decades^69^. Given their low cost and easy fabrication, microneedles have been widely used in single-cell surgical methodologies. Microneedles attached to a micromanipulator have been used for several cell surgical tasks, such as cell biopsy^70^, cell injection^71^, cell cutting^72^, patch clamping^73^ and organelle removal and transfer^74^. Cell injection is the procedure of injecting external materials into a cell by a microneedle-based microinjection system^75^. A microneedle controlled by a micromanipulator perforates the cell membrane with its small and sharp tip and then injects materials into the cells, as illustrated in Fig. 3a. To restrict the transmission of inherited mtDNA diseases, the pronucleus of diseased women is injected by a glass microneedle to enucleate donor eggs, which may lead to offspring with healthy mtDNA^76^. Although this assisted reproduction strategy offers a healthy embryo, it cannot be utilized after birth or on somatic cells^77^. Another use of glass microneedles is patch clamping. Patch clamping is a method of studying the ionic currents and electrical behavior of individual isolated living cells and cell membranes. Micropipette tips coated with electrodes are placed on the cell surface, and a part of the cell membrane is aspirated to create a small opening for measuring the current levels.

To minimize the physical damage to cells due to penetration, several nanoneedle-based systems have been developed. Recently, a scanning ion conductance microscopy-based nanobiopsy system was developed to extract femtoliter samples of intracellular content by glass micropipette to analyze mRNA and mitochondrial DNA. This technique utilizes electrowetting to take up samples of cytoplasm into a glass nanopipette and then applies high-throughput sequencing technology to analyze the biopsied cellular material^78^, as shown in Fig. 3b. Similarly, researchers developed DEP-based nanotweezers^79^ made of two closely spaced electrodes with gaps as small as 10–20 nm to trap DNA and proteins in a dielectrophoretic manner, as shown in Fig. 3c.

Researchers have used this technique to extract nucleic acids from living cells for gene expression research without affecting cell survival, in addition to trapping single molecules. They also demonstrated the extraction of a single mitochondrion by using nanotweezers. To minimize cell surgery damage, researchers have developed a carbon nanotube-based endoscope that can interrogate cells, transfer fluids, and conduct optical and electrochemical diagnostics at the single-organelle level^40^ (Fig. 3d). The endoscope, which is created by inserting a multiwalled carbon nanotube (length, 50–60 µm) into the tip of a glass pipette, has a spatial resolution of ~100 nm and can reach organelles without disturbing the cell. When the nanotube is loaded with magnetic nanoparticles, the endoscope may be moved remotely to transfer nanoparticles and attoliter quantities of fluids to and from specific places. Given that the endoscopes are placed on ordinary glass micropipettes, they easily fit common equipment, opening up a wide variety of possibilities for minimally invasive intracellular probing, medication administration, and single-cell surgery.

The abovementioned micromanipulation procedures also necessitate skillful operation by a technician, which is time-consuming. The technician can inject only one cell at a time, restricting throughput. The development of technologies such as automation equipment and robotic systems in recent years has significantly increased the therapeutic efficiency of microinjection technology, although significant throughput and scalability need to be achieved (Fig. 3e, f)^22^. Automation of these complex single-cell surgical tasks is in high demand to reduce the probability of contamination resulting from human errors, labor-intensive work, process uncertainty, and variable outcomes. We developed several robot-aided single-cell surgery systems to automate organelle-level complex surgical procedures^17,19,21,60,63,64,80,81^. These automated systems were developed to reduce the human error involved in the precision surgery of single cells. Recently, we developed a robotic single-cell biopsy system for mitochondria and nuclei, as shown in Fig. 3g^19^. A microfluidic chip was used to trap single cells, and a glass micropipette was used to biopsy mitochondria from single cells in an automated way with high precision. Similarly, mitochondria extracted from a single cell can be further transferred to another cell using a microneedle-based mitochondrial transfer system, as shown in Fig. 3h^80^. Precise cell injections were performed with an initial 3D reconstruction of a cell to determine the specific target location, such as the nucleus inside the cell^82^.

Biomedical applications of microneedle-based single-cell surgery methods are limited by the throughput and physical damage to cells caused by microneedle penetration into the cell as the cell size decreases relative to the microneedle tip. For large cells, such as oocytes (100 µm diameter), a large number of mitochondria can be injected relatively easily by using a micropipette with a tip size of approximately 5–10 µm. However, for small cells, such as somatic cells ~20 µm in diameter, the size of the micropipette tip cannot be larger than 1 µm during mitochondrial injection to keep the recipient cell alive^83^. When injection pressure is applied, injected materials may become stuck at the tip and clog the micropipette tip, which can lead to low efficiency of microneedle-based mitochondrial injection. Therefore, the glass microneedle-based mitochondrial injection method has several limitations, including clogging of the glass micropipette tip^84^, physical damage to the cell, and limited opportunity for the repeated injection into the same cell.

To address the above problems, the nanothermal blade method was developed^85^, which uses a micropipette with a diameter of >3 µm to prevent clogging, as shown in Fig. 3i. This method uses pulsed laser-induced bubble cavitation to open holes in the cell membrane and then employs a synchronized fluid to pump mitochondria. Moreover, in this method, the injection efficiency of mitochondria is only 2–3% because the micropipette is only placed on the surface of the cell membrane and is not tightly wrapped inside the cell membrane. Therefore, during the injection process, mitochondria may be unable to enter the cell.

Integrating microneedles onto traditional patches has shown the ability to effectively deliver a range of drugs, including proteins, antibodies and vaccines^86^. Most microneedles have tips tens of microns in size and cannot accurately target individual cells, resulting in uneven and invasive delivery of plasmids or other macromolecules. To address this problem, nanoneedles with sharp smaller ends (<100 nm) were fabricated. They provide high precision and minimally invasive manipulation at the single-cell level. Kim et al. demonstrated the use of nanowire (NW) arrays to penetrate cells, allowing gene transfer into mammalian stem cells^87^. When NWs enter cells or cells are placed on NWs, molecules attached to the NW walls may dissociate and enter the cytoplasm (Fig. 3j)^88^. In this way, biomacromolecules can be transferred into cells without chemical modification or viral packaging. Shalek et al. used chemical vapor deposition techniques to create vertical NWs^89^. In Shalek’s study, HeLa cells with 1,1-dioctadecyl-3,3,3,3-tetramethylindodicarbocyanine (DID)-tagged membranes were placed on green fluorescently labeled NWs, and the permeation process was observed^86^. This technology has the potential to deliver siRNA, peptides, DNA, proteins and impermeable inhibitors into sensitive cell types such as neurons and immune cells. However, the NWs were unable to enter the cell immediately after contact^90^. Although the NWs developed above allow high-throughput cell injection, this approach may not enable precise dose injection. Furthermore, active injection or control of the penetration time of NWs still needs to be addressed, as direct penetration of cell membranes by mechanical structures often leads to permanent cell damage. This situation requires continuous innovation and improvement of micro/nanoneedle preparation methods and materials to provide high delivery efficiency while minimizing damage to cells.

### Untethered microtools for single-cell surgery

#### On-chip microtools

**Fig. 4:**
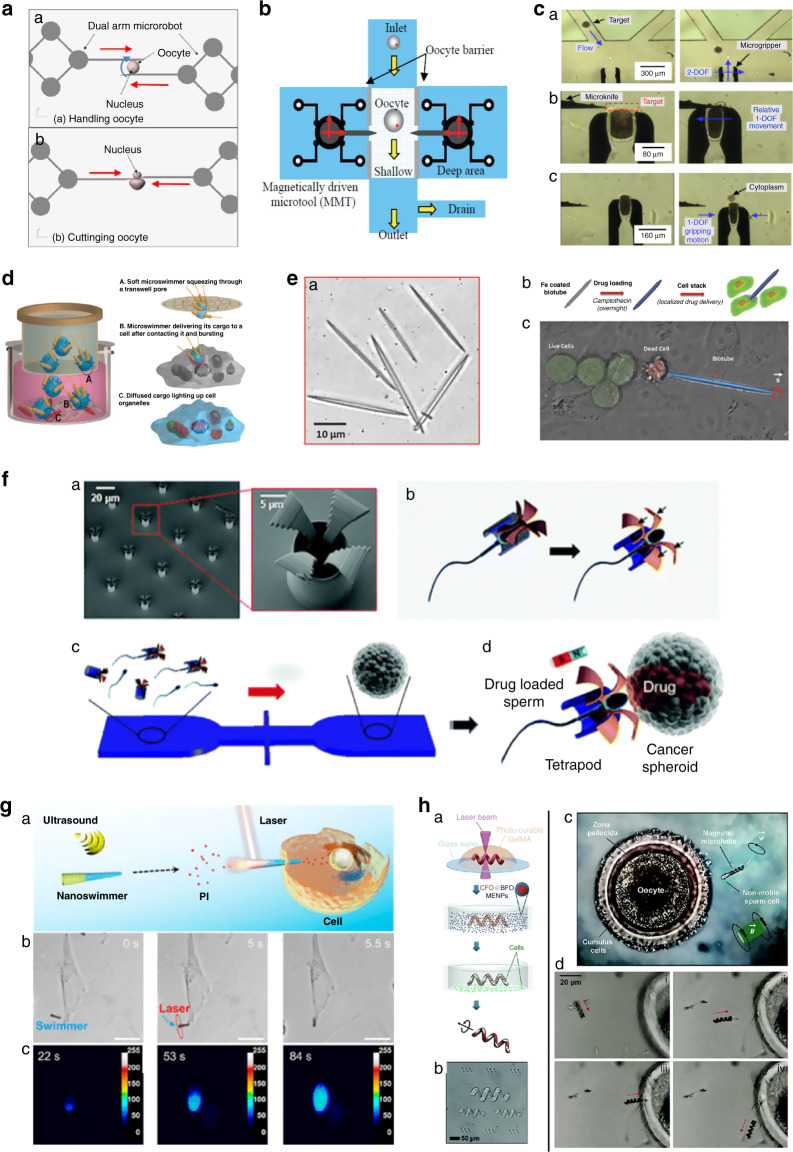
Untethered micro/nanotools for single-cell surgery. **a** Cell cutting by a magnetically controlled dual-arm robot. Copyright 2010, IEEE^99^. **b** On-chip enucleation of the oocyte. Copyright 2009, IEEE^100^. **c** Enucleation of an oocyte by using a microgripper and a micro knife. Adapted with permission^101^. **d** Soft bacteria-driven microswimmers based on microemulsions for active cargo delivery. Adapted with permission^103^. **e** Dual-action biogenic microdaggers for single-cell surgery and drug release (Medibots). Adapted with permission^104^. (b) Schematic and (c) experimental representation of medibots for cancer cell killing. **f** Targeted drug delivery at the cellular level using a sperm-hybrid micromotor. Adapted with permission^187^. (a) SEM images of a printed tetrapod microstructure array. (b) Diagram depicting the mechanical release mechanism. (c) The microfluidic chip for drug-loaded sperm transport and delivery is shown schematically. (d) An image sequence depicting the sperm release process when the arms collide with HeLa cells. **g** Photomechanical poration of single-cell membranes using a gold nanoshell-functionalized polymer Nanoswimmer. Adapted with permission^106^. (a) Schematic cell poration of AuNS-functionalized nanoswimmers after NIR laser exposure. (b) Time-lapse pictures of nanoswimmers moving toward a HeLa cell in an acoustic environment and perforation with NIR irradiation. The blue dashed line represents the acoustic driving route, while the red circle represents the laser point area. (c) Time-lapse colormap pictures of the dynamic intracellular distribution of fluorescence intensity after NIR irradiation of the nanoswimmers. Scale bars are 10 µm long. **h** Untethered microswimmers for cell delivery for disease therapy. Adapted with permission^188^. (a) Biodegradable helical microswimmer manufacturing method using CoFe_2_O_4_ (CFO, core) and BiFeO_3_ magnetoelectric nanoparticles. (b) Optical image of helical GelMA microstructures created by two-photon polymerization (2PP). (c) Micromotors that transport sperm for assisted fertilization. Adapted with permission^107^. (d) Remote collection and transportation of immotile sperm to an egg for fertilization utilizing a magnetic microhelix. Coupling of microhelix and immotile sperm (i), sperm transportation (ii), sperm approach to the oocyte membrane (iii), and sperm release (iv)^106,107^.

Several microfluidic-based micromanipulation platforms for untethered control of surgery tools have been developed to perform contamination-free automatic single-cell surgery^91–96^. Animal cells were first softened by chemical treatment, and then two orthogonal channels controlled by an external magnetic field were used to cut the cell in half. The half of the cell containing the nucleus was used for cloning^97^. A microfluidic device integrated with thermopneumatic actuators was used for controlled cell lysis^98^. Upper channels were used for the introduction of cells, and lower lysed cells could be collected at lower channels. An oocyte was cut by a magnetically manipulated cutter to remove its nucleus, as shown in Fig. 4a^99^. On-chip nuclear removal (enucleation) was performed using a magnetic field-controlled microrobot to increase the speed of enucleation^100^ (Fig. 4b). Similarly, after grasping an oocyte with a microgripper, the cytoplasm of the cell was extruded with a micro knife, as shown in Fig. 4c^101^. Several other contamination-free, automated and high-throughput microfluidic-based enucleation procedures of oocytes were performed^92,95^. Although the above microrobotic-based platforms have been shown to be efficient surgical techniques, they have not been used for surgery of somatic cells because of the severe damage caused during cell dissection.

#### Untethered micro/nanotools

To perform untethered surgeries on single cells, several untethered micro/nanotools and systems have been established recently^102^. Bacteria-driven microswimmers have been created that possess active locomotive and bacterial sensing capabilities with the desirable enclosure and viscoelastic characteristics of a soft double-micelle microswimmer for active transport and transfer of cargo to living cells (e.g., genes)^103^ (Fig. 4d). This in vitro model demonstrates that soft microswimmers offer a promising opportunity for biomedical applications for active and/or targeted transport and delivery in in vitro models (e.g., organ-on-a-chip devices) and stagnating or low-flow liquid areas in the body. Researchers have also reported “dual-action microdaggers,” which are plant-derived biogenic micromotors that can produce a cellular incision followed by drug release, enabling extremely targeted drug delivery^104^ (Fig. 4e). The biogenic hybrid micromotor with “dual action” (e.g., cell microdrilling and drug release) enables noninvasive surgery with the single-cell targeting accuracy, as well as the additional benefit of drug release. Schmidt and colleagues created biohybrid sperm micromotors (Spermbots) as a targeted drug delivery system to treat female reproductive system disorders^105^ (Fig. 4f). 2PP 3D printing was utilized to create magnetic tubular microstructures for transporting motile sperm that serve as a propeller or transporter and anticancer medication carrier. Drugs such as doxorubicin (DOX) can be loaded onto sperm, giving good encapsulation, transport, and transfer stability. These spermbots can be magnetically directed to swim within the in vitro tumor model and release sperm to distribute DOX locally via fusion of cell and sperm membranes. Spermbots can also be potentially used in the human physiological environment because of their biohybrid design. Spermbots may have promising applications in gynecological treatments, for example, the treatment of ovarian cancer, cervical cancer, and other gynecological diseases, while avoiding harmful side effects of drugs on healthy tissues and/or organs. Researchers have developed an ultrasound-driven and near-infrared (NIR)-operated nanoswimmer that can puncture the membrane of a cancer cell^106^ (Fig. 4g). These nanoswimmers were able to move efficiently and controllably toward target cells under manipulation by an acoustic field. According to the experimental and theoretical findings, the immediate photothermal action generates sufficient photomechanical force to perforate the membrane of the cell. Such NIR-assisted nanoswimmer-enabled cell membrane poration has several advantages over conventional chemical and physical cell poration techniques, including active, fast, and precise targeting for single-cell surgery. Thus, this technique has tremendous potential for a number of biological applications, including gene transfer and fertilization. Artificial motorized sperm cells were created—a novel kind of hybrid micromotor in which customized microhelices serve as motors to help sperm cells with motion deficiencies in completing their usual function^107^ (Fig. 4h). This robot can collect, transport, and release single immotile living sperm cells in fluidic tubes. Although certain difficulties remain on the path to successful fertilization using artificially motorized sperm, the promise of this new method for assisted reproduction is already evident.

Although the above untethered micro/nanomanipulation platforms present several advantages, such as contamination-free and precise surgery without the use of expensive micromanipulators, the precision, efficiency, and throughput of these micromanipulation systems are low. Cell manipulation systems such as the pressure-driven injection system (Mitopunch) can produce hundreds of modified cells simultaneously^108^. By applying force on a mechanical plunger, thousands of cells can be injected with foreign substances at once, which can greatly improve the throughput of cell surgery.

Advances in medical robotics have the potential to improve current medicine and overall quality of life^5^. As these robotic platforms become more compact, they open the door to new applications of precision medicine. The unconstrained micro- and nanorobots used for precision medicine continue to face technical, regulatory, and commercial difficulties that must be overcome before they can be widely used in clinical settings. Nonetheless, recent translation from proof-of-concept to in vivo studies suggests their promise for precision medicine and personalized therapy^109^. The shrinking of robotic platforms provides the potential to improve patient care and diagnosis. These small robotic doctors may offer us access to hard-to-reach areas of the body, as well as the ability to perform a variety of medical treatments. Despite advances in medical micro/nanorobotics over the past decade, one of the unmet requirements and important hurdles in the field is the translation of these instruments into broad clinical applications, which is still a long way off.

Microrobots have a wide range of applications in precision medicine, including drug delivery, biologic delivery, gene delivery, and live cell delivery; surgical tools for biopsy, tissue penetration, intracellular delivery, or biofilm degradation; diagnostic tools such as physical and chemical biosensors or isolation tools; and optical, ultrasonic, magnetic and radionuclide imaging tools. Targeted delivery is the most established use, and current efforts are largely focused on animal testing. To understand the dynamics of micro/nanorobots and maximize their efficiency and capabilities, imaging must be used in conjunction with delivery, manipulation, or diagnostics. Current nano/microtool research shows that the gap between precision medicine and micro/nanorobotics has been narrowing. Nonetheless, each application presents unique barriers to clinical translation. Micro/nanorobots must operate in hard-to-reach areas of the body for delivery and surgical applications; therefore, recovery/degradation procedures are critical to ensure that they do not endanger the health of patients^110^. Furthermore, before micro/nanorobots can be used in therapeutic settings, they must overcome safety, technical, regulatory, financial and commercial hurdles. Although there is still a long way to go, the use of microrobots in precision medicine can definitely enhance diagnosis and treatment, leading to improved quality of life for patients. Microrobots may aid in precision medicine while also reducing the cost and pain of major surgical procedures.

## Control methods

Cells and their intracellular components are small^111,112^ and fragile^113,114^, and they are distributed randomly in the medium^112,113^ with irregular shapes^115,116^. Operation failure and damage are likely to occur under inappropriate manipulation control. In addition to developing cell surgery tools, current studies face the following two challenges: how to achieve precise position localization of targeted intracellular specimens for successful cell surgery micromanipulation and how to regulate cell damage for a high survival rate. However, localizing operation positions can meet unignorable difficulties resulting from the complexity of intracellular environments and culture media, such as counterpart cells and auxiliary manipulation tools^117–119^. Random distribution of biological targets requires tedious height adjustment of optical lenses to ensure precise detection of targets^120^. Moreover, the imaging quality is deteriorated by imaging blur and noise during such optical observation, making the boundary of the targeted object unclear^121^. Therefore, visual servoing should be carried out based on the position feedback from advanced imaging processing. For the second challenge, cell morphology is usually adopted to estimate cell survivability, where dead cells often exhibit broken surfaces and lose adhesion to the substrate (for adherent cells). Fluorescence is another reflection of cell viability because cells undergoing damage exhibit low fluorescence intensity. For example, calcium flux^78^ and membrane potential probes^122^ are widely used for cell and mitochondrial function tests, respectively. However, these observation methods are qualitative and not accessible for cell damage regulation control. Force provides a quantitative parameter for cell viability measurement; cell damage from the interaction of manipulation tools can be monitored during micromanipulation; and the measured force signal can be adopted for the robotic manipulation control of such tools^123^. In practice, precise force sensing is difficult because of the small amplitude of the response signals of biological targets^124–126^ and high-level environmental noise^7,127,128^. With the help of state-of-the-art sensing instruments and postcomputation procedures, force-based control during manipulation is achievable. Thus, the success rates of cell surgery tasks can be improved, and operation damage can be reduced.

### Visual servoing and controls

Successful robotic surgery manipulation control heavily relies on the localization of biological targets and manipulation tools. Targeted objects can be imaged and sampled with optical microscopes, such as wide-field fluorescence microscopes and confocal fluorescence microscopes. Out-of-focus blurs and imaging noise are inevitable in original sampled images and may produce inaccurate contours and localization of targeted specimens. Therefore, computational image processing methods are required to restore the morphology, position, orientation, and other operational details of targets.

**Fig. 5:**
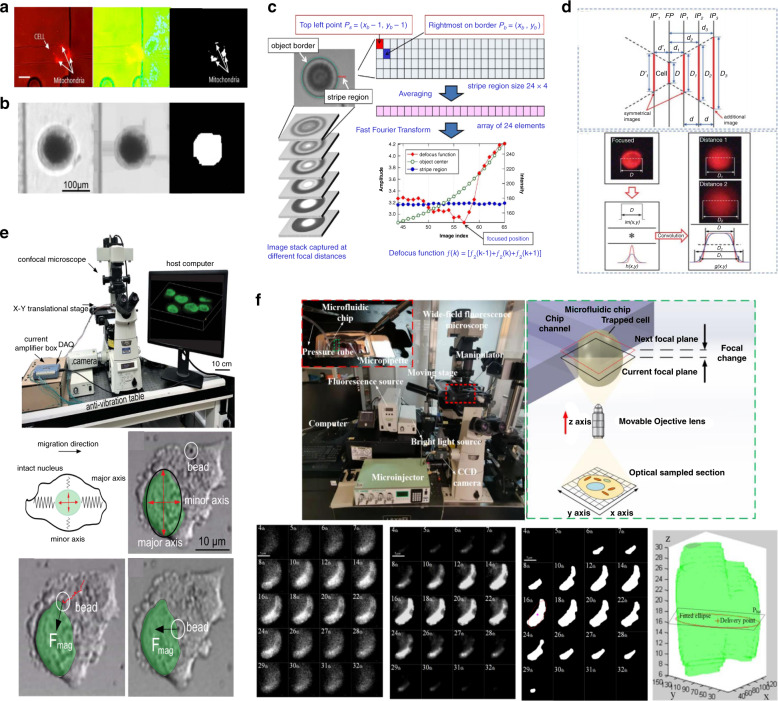

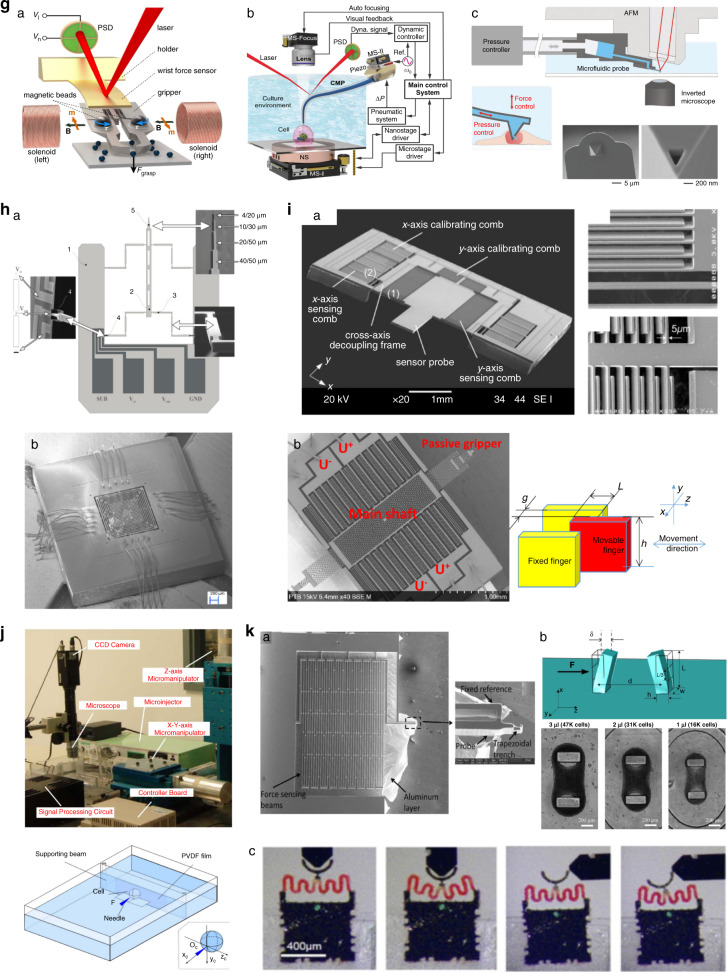
Visual and force sensing and control methods. **a** Adaptive Otsu thresholding process of an oocyte^19^. **b** Adaptive thresholding process with HSV range values^133^. **c** Depth from focus (DFF) methods based on the border intensity variation. Copyright 2015, IEEE^140^. **d** Depth from defocus (DFD) methods based on the Gaussian distribution model. Copyright 2015, IEEE^143^. **e** Confocal microscope-reconstructed 3D model of bladder cancer cell nuclei for mechanical measurement. Adapted with permission^145^. **f** Reconstruction process of an HDFn nucleus with wide-field microscopy^17^. **g** Atomic force microscopy (AFM) with different force sensing probes. (a) Customized magnetic microgripper for microassembly. Copyright 2020, IEEE^151^. (a) Cantilevered micropipette probe for microassembly^152^. (b) Nanofluidic channel probe for microinjection. Copyright 2020, IEEE^189^. **h** Piezoresistive force sensors with delicate sensing structures. (a) Piezoresistive force probe with small-scaled beams. Adapted with permission^156^. (b) Piezoresistive force sensor with folding springs. Adapted with permission^157^. **i** MEMS capacitive force sensors with differential combs. (a) Capacitive force probe with gap-variant combs. Adapted with permission^162^. (b) Capacitive force probe with area-variant combs. Adapted with permission^163^. **j** PVDF piezoelectrical film for cell indentation force measurement. Copyright 2011, IEEE^164^. **k** MEMS optical force sensors. (a) Optical probe with micromachined springs. Copyright 2011, IEEE^168^. (b) Flexible pillars for cardiac tissue construction. Adapted with permission^169^. (c) Untethered microrobot integrated with a vision-based sensing probe. Copyright 2018, IEEE^170^.

Threshold binary is a widely adopted segmentation method to obtain distinct boundaries of targeted objects, where sampled features are binarized into bright spots or black backgrounds in terms of a threshold value. To smooth the sampled images, Gauss^80^, Median^129^, Wiener^130^, and Hilbert filters^131^ were adopted before the segmentation process. Thereafter, cell contours can be highlighted with edge detectors, such as the Canny edge detector^80^ and the Sobel operator^132^. Cells or other biological targets are normally covered by other devices and similar objects. Direct segmentation with a constant threshold value can hardly distinguish them from one another and may erase target boundaries near similar targets. Therefore, some adaptive thresholding processes were proposed that utilize identification factors to adjust the threshold value of each image. The hue, saturation and value (HSV) range value is proposed to divide the region of interest into four ranges, and the specific threshold value can be obtained by applying these values on the HSV plane image^19^, as shown in Fig. 5a. Figure 5b shows an Otsu thresholding process whose threshold value is derived from the area and roundness of the targets^133^. The segmented boundary can be used to locate the target object and facilitate manipulation, and the success rate of automated mitochondrial extraction and oocyte enucleation can reach 60%^19^ and 93.3%^133^. Notably, these segmentation approaches can extract features only at a fixed imaging depth, and the targeted specimen is assumed to be a regular sphere, whose center is usually localized as an operating position. For targets with irregular morphology, the appropriate operation position should be selected through spatial information^134^.

Recently, some template-based depth acquisition methods have been developed, where a focal image of the biological targets or surgery tools is sampled and set as a template for comparison with sampled images at different optical depths. The focal imaging depth can be estimated when the pixel differences between the sampled images are minimized^135,136^. However, external disturbances, noises, and imaging errors can produce inaccurate depth when the imaging field moves greatly. To alleviate this problem, some optimized template-matching criteria have been proposed based on template similarity score^137,138^, three frames^18^, and centroid coordinates^139^. With the depth information obtained, the microinjection efficiency is greatly improved, with experimental results showing that more than 1500 cells can be processed automatically within 1 hour^18^. However, template selection is manual and labor intensive, and a new template is required when targets change. Imaging practices have shown that the light intensity of sampled targets follows an organized distribution model. Many attempts called depth from focus (DFF) methods^140,141^ have been applied to locate the focal depth where a rapid change in light intensity occurs, as shown in Fig. 5c. Depth from defocus (DFD) methods^142,143^ are efficient because they can estimate current depth by comparing neighboring defocused images, as shown in Fig. 5d. However, DFF and DFD estimations suffer from low depth resolution and become inaccurate if the target’s height is smaller than the sampling depth intervals.

For small biological objects with irregular morphology and random distribution, 3D image reconstruction of biological targets can be carried out to provide a comprehensive geometric analysis. Most out-of-focus emissions can be physically filtered by a pinhole aperture in a confocal microscope; the 3D morphology of endothelial cells^144^ and bladder cancer cell nuclei^145^ have been reconstructed for applications of cell microinjection and biophysics measurement, as shown in Fig. 5e. Wide-field microscopes are extensively used. The in-plane features of sampled images contain out-of-focus blurs projected from adjacent depths and noise. Noise-regulated maximum likelihood estimation deconvolution was proposed to eliminate such deteriorations^82^. After filtered segmentation, the deconvoluted images of the neonatal human dermal fibroblast (HDFn) nucleus can be reconstructed into a 3D model, and an optimal position can be obtained under volume-based geometric analysis to improve nuclear delivery accuracy and efficiency, as shown in Fig. 5f^17^. The 3D reconstruction information can greatly improve the operation accuracy while reducing the damage to biological objects. Specifically, the success rate of mitochondrial extraction experiments is 20% higher than that of traditional 2D methods, preventing the cell destruction that usually occurs in traditional methods. The 3D reconstruction information was also applied to automated intracellular delivery. The success rate of nucleus delivery increased to 71%, well above the value of 48% obtained in 2D experiments. More than 85% of processed cells showed strong biological activity when incubated 24 h after delivery. However, the computational complexity and image sampling of 3D reconstruction require a relatively long processing duration, and real-time reconstruction is difficult to realize. Thus, a tradeoff exists between control accuracy and manipulation throughput.

With such visual localization feedback, the robotic manipulation controller manipulates surgery tools or biological objects to minimize the error between their origin and destination positions is minimized. Differentiation terms of position error derived from the velocity of the manipulated objects are essential for motion control. However, direct measurement of such velocity not only requires complicated hardware schemes but also suffers from system disturbances and external disturbances. Some velocity observers and estimators were proposed to compensate for the error caused by inaccurate information of the camera position. An uncertainty and disturbance estimation-based observer was proposed to estimate cell angular velocity for cell orientation control^146^. A model-free disturbance observer^147^ and high-gain observer^148^ were developed to estimate the velocity and state errors for the control of microrobots in blood vessels. With such observers, a simple saturated proportional–integral–derivative (PID) controller was proposed for the asymptotic regulation of cell rotation^149^. A feedforward plus PD feedback controller was proposed for high-throughput cell microinjection^132^. In addition, several sliding mode controllers were developed to manipulate microneedles for intracellular delivery^17^ and biopsy^150^. By using such advanced motion controllers with precise visual feedback, the operation accuracy and success rate can be improved.

### Force sensing and controls

Current surgical tools manipulate cells and other biological objects by either physical interaction or field-driven forces; the interaction forces between them unavoidably do great harm to the biological viability of the cells if a certain value is exceeded. Therefore, interaction force control is significant for damage regulation in robotic cell surgery manipulation. Nevertheless, the amplitude of the force signal during manipulation is small (less than a few hundreds of micronewtons), and the signal accompanied by strong background noise from the complicated environment of living cells, which puts force sensing techniques with high sensitivity and signal-to-noise ratio (SNR) into urgent demand.

AFM has a very high force-sensing resolution at the piconewton level. The forces between the probing tip and a sample surface cause cantilever deflection, which is detected by an optical system composed of a laser and a photodetector. Customized magnetic microgrippers^151^, cantilevered micropipette probes^152^, and nanofluidic channel probes^152^ have been integrated into AFM systems for applications in microassembly, microinjection, and intracellular delivery, as shown in Fig. 5g. In addition to the labor-intensive optical alignment and adjustment, signals from AFM are measured by the outer reflection of the laser beam, which can result in inaccurate force measurements when reflected light transmits through the culture medium^153,154^. MEMS sensors, which take advantage of task-oriented design, high sensing performance, and easy mass production, have been widely used for force sensing in robotic cell surgery manipulation. On the basis of the resistance change resulting from exerted forces, the piezoresistive force sensor normally produces stable force signals with relatively low resolution at the micronewton level. A soft flexure beam cell microinjector embedded with a piezoresistive force sensor has been developed, which has a force resolution of hundreds of micronewtons^155^. Piezoresistive probes with a resolution of tens of nano-Newtons have been proposed, which typically have flexible beams^156^ or springs^157^ with widths less than 5 µm and lengths greater than 1000 µm to achieve high stress concentrations, but the cost is significantly difficult to fabricate, as shown in Fig. 5h. MEMS capacitive force sensors can measure the forces when external forces produce transverse or longitudinal movement of parallel electrodes. With comb-structured capacitive electrode plates, they can incorporate multiple sensing arrays within one unit, thereby achieving high sensing sensitivity. Although a narrow air gap between the comb plates is preferable for higher sensitivity, capacitive sensors are much easier to fabricate than piezoresistive sensors because the comb plates are typically less than a few hundred micrometers long. Several capacitive force sensors using differential comb capacitors have been proposed and can measure nanonewton-level forces^158–161^, as shown in Fig. 5i. However, the fabrication consistency deteriorates as the number of combs increases. Furthermore, capacitive sensors are susceptible to high cross-coupling and low linearity owing to unavoidable parasitic capacitances^162,163^; thus, a complicated readout circuit is needed. Piezoresistive and capacitive force sensors are mainly integrated with surgery tools for measuring the forces exerted by such tools. Piezoelectrical sensors can be mounted at the targeted cell side, and loaded forces can compress or stretch the film and produce corresponding electrical voltage. Polyvinylidene fluoride (PVDF) film can be adhesively bonded to a cell bracket for cell indentation force measurement^164,165^, as shown in Fig. 5j. Considering the macroscopic film size, PVDF piezoelectric sensors are easy to fabricate, while their force resolution is relatively low at the micronewton level^166,167^. In addition, they are not applicable for force measurement in intracellular manipulation because they can hardly measure forces inside the cytomembrane; thus, their spatial resolution is greatly limited. The optical sensor has large sensing ranges for observing the displacement of the sensing apparatus with known stiffness, and its resolution is comparable with the pixel size of the imaging microscope. Micromachined springs^168^, flexible pillars^169^, microrobots^170^, and other types of optical sensors have been developed to measure intracellular forces under microscopic fields, and they can be easily integrated into untethered microtools, as shown in Fig. 5k. However, optical observation suffers from imaging blurs and noises, which can lead to difficulty in distinguishing the sensing structure from backgrounds, especially for cases that are not at the focal plane^171^. To improve measurement accuracy, optical sensors usually use flexible materials or low-rigidity structures. In addition to increased fabrication difficulty, geometric deviations can create stiffness inconsistencies between fabricated sensors, so optical sensors require careful performance calibration^172^. In general, force sensors should be selected in terms of micromanipulation tasks and their own characteristics. Sensing resolution and signal conditioning should be evaluated first, especially for intracellular organelle micromanipulation tasks that require sensing resolution higher than the nanonewton level. Furthermore, the convenience of integration with manipulation tools should be considered.

Force feedback control can be simultaneously initiated once force signals are obtained from appropriate sensors. Direct force control and indirect force control are the two main categories of manipulation force control. For the indirect type, the forces exerted on cells are typically converted to the position of manipulation tools depending on optical observations, guaranteeing a smooth switch. An impedance controller^173,174^ and a PID controller with optimal control functions^175^ have been proposed for maintaining the desired force and displacement amplitude. A two-loop control framework controller improves the force regulation performance, where a force tracking nonlinear controller with measured feedback from PVDF piezoelectrical sensors is an external loop for the internal impedance control loop^176^. The penetration force of zebrafish embryos can be adjusted to 216 µN, and the relative root mean square error (RMSE) of force tracking is approximately 0.37. Given that the force is estimated from optical observations, indirect force control suffers from inaccurate control parameters. It may be unreliable for complicated manipulation tasks such that target deformation is undetectable. Direct force controllers move manipulation tools with straightforward force feedback from integrated sensors once they arrive at the target position under position control. Some PID/PD controllers based on incremental function^177^ and model-compensated prediction schemes^178,179^ have been proposed to regulate cell loads, where the maximum force tracking error is limited to 90 µN^177^ and the regulation resolution reaches 50 µN^178,179^. The force controller takes charge when the measured force exceeds a threshold value; thus, a stable switch between position and force control is strongly needed. A weight-based fusion approach with introduced weight coefficients in the control variable has been proposed to fuse the two position control variables and force in an overlap interval of embryo microinjection manipulation^180^. A revised noise-insensitive extended high gain-observer^181^ and an event-based switch criterion^182^ have been proposed to minimalize the switch overshoot for contact force regulation. Compared with the traditional PID controller, the force overshoot is greatly improved by 49%^179^, and the force tracking RMSE is reduced by 34.2%. From the aforementioned illustration, it is clear that force regulation controllers can greatly decrease the cell deformation resulting from tool interaction; cell breakage, bursting, and other major damage can be prevented as well.

## Discussion and conclusion

Micro/nanoscale biological cell operations, such as single-cell surgery, have recently become important due to their key biological applications in precision medicine. Single-cell surgical approaches, such as cell injection, cell biopsy, and the extraction and transfer of cell organelles from a single cell, have key biological applications, such as studying diseases and their causes, in greater depth. The development of tools and methods for single-cell surgery can play an important role in the treatment of diseases, such as aging and neurodegenerative disorders, epilepsy, and type 2 diabetes. In this article, the tools and systems for single-cell organelle surgery are summarized in detail. The current challenges and drawbacks of existing methodologies are also highlighted.

As mentioned above, several single-cell surgery systems, such as single-cell injection and organelle manipulation systems, have been developed in recent years. Single-cell organelle-level surgery or manipulation is more challenging than cell injection for the following reasons. First, in the case of cell injection, a precise and specific location is generally not mandatory inside the cell; in cell organelle manipulation or surgery, the tooltip must be positioned at a precise location of the organelle in the 3D space of the cell. Second, unlike organelle biopsy, cell fluidic injection can be completed with a tip size of 0.1–1 µm, which is an acceptable range to keep the cell alive after injection. However, organelle biopsy or transfer requires a relatively large tooltip size depending on the organelle size, which can easily damage the cell. Third, in organelle manipulation, the cell-holding power must be readjusted during tool withdrawal. Therefore, introduction of the tooltip at a precise position inside the cell, use of a relatively larger tip, and suitable control of the cell during organelle biopsy or removal cause single-cell organelle manipulation or removal to be more complex than cell injection.

Typically, enucleation methods are performed on cells 50–1000 µm in diameter. The removal and transfer of subcellular components from small biological cells (<25 µm in diameter), such as human mesenchymal stem cells (MSCs), are more complicated than those from large biological cells, such as zygotes. The core challenges in small-cell surgery are precise micropipette or cell positioning and the prevention of irreversible physical damage to these sensitive cells during surgery.

Notably, the direct penetration of cell membranes through mechanical structures often results in irreversible cellular damage. Solving this problem requires continuing innovation and the development of micro/nanoneedle fabrication techniques and materials to achieve high delivery efficiency while preventing cellular damage. Furthermore, current single-cell surgery or modification methods are primarily limited by throughput and efficiency. To further expand the use of modified cells for in vivo or clinical applications, which require millions of engineered cells, the throughput of single-cell surgical approaches needs to be significantly increased. Less invasive micromanipulation systems, such as pressure-driven BLAST (Fig. 2h)^67^ and Mitopunch^108^, can generate hundreds of modified cells simultaneously.

With the rapid development of precision engineering and medical technology, the use of engineering methods for surgical intervention on single biological cells is an emerging technology in the field of medical applications. This development is in line with the trend of expanding modern therapies from the organ level to the cellular level to prolong patient survival. Although existing cell modification engineering methods exhibit many advantages, such as high precision and selectivity in generating functionally altered cells, these methods have relatively low throughput compared to traditional chemical or biological methods in cell therapies. There is a high demand for the development of automated table-top microfabrication systems capable of efficiently and rapidly processing tiny biological samples. Automated micromanipulation systems can incorporate manipulators, such as vision-guided and robot-driven micropipettes and OTs, and microfluidic chip devices for automated cell processing. Dynamic image processing and robust control techniques can be used to realize the large-scale cell modification process required for clinical therapy. The success of these systems will validate the feasibility of the large-scale production of functionally altered cells by integrating robotics and fabrication techniques into cell manipulation. The successful production of therapeutic quantities of characteristically modified cells will have broad and diverse applications in biopharmaceuticals, gene and cell therapy, and tissue engineering.

Table 1 summarizes and compares different technologies for single-cell surgery.

**Table 1:** Comparison of single-cell surgery tools and methods.

Method	Surgical methodology	Advantages	Disadvantages	Applications
Laser	Argon or femtosecond laser based methods^50–53,55,183^	High resolution	Low throughput, membrane reclosing is difficult to control	Cell membrane cutting, organelle disruption, cell fusion, cell lysis
	Blast laser^67^	High throughput, high cell viability, high efficiency	High cost	Cell injection, membrane disruption
	OT-assisted surgery^21,57,64,66^	Noninvasiveness, high efficiency, automation, and high accuracy	Low throughput, low power, high cost	Cell manipulation, organelle extraction or transfer, and cell rotation
Untethered physical tools	Magnetically controlled on-chip tools: i) Manual control^98^. ii) Automated control^91–97,101^	Contamination free and high throughput	Low accuracy and dependent control of targets	Enucleation, cell lysis, and drug delivery
	Magnetic controlled micro/nanotools^95,102,104–106^	Contamination free and high throughput	Low accuracy and independent control of targets	Cell lysis and drug delivery
	Ultrasound controlled^105^.	Contamination free and high throughput	Low accuracy and independent control of targets.	Cell lysis and drug delivery
Micro/nano needles	Glass microneedle i) Manual control^78^ ii) Robotic control control^17–19,80–82^	Easy fabrication, cost-effective, flexibility of tip size, and high throughput with robotic control	Low throughput if manually controlled	Cell membrane cutting, organelle biopsy, organelle transfer, and cell injection
	AFM^28^	High resolution, and noninvasive	Low throughput, high cost	Cell injection, cell surface analysis
	Photothermal nanoblade^85^	Enables large cargo injection in single cells	Low throughput and low efficiency	Mitochondrial transfer, cell injection
	Array microneedle^184^	High throughput, noninvasiveness, and high efficiency	Less accuracy and difficult to inject large cargo in cells	Nanoparticle and molecular injection in a large number of cells simultaneously
